# Prevalence of Infant Shaking Among the Population as a Baseline for Preventive Interventions

**DOI:** 10.2188/jea.JE20150321

**Published:** 2016-01-05

**Authors:** Noriko Kato

**Affiliations:** Department of Early Childhood Care and Education, Jumonji University, Saitama, Japan; 十文字学園女子大学人間生活学部

**Keywords:** shaking, smothering, shaken baby syndrome, prevalence, population

## Abstract

乳児は生後2～3か月までは、特に理由もなく泣き、なにをやっても泣き止まないほど激しく泣くが、正常な経過であり、次第に収まっていく。こういった泣きに対して、より耐性を持ちがたい親は、イライラしたり、無理に泣き止まそうとしたりして、ゆさぶりや口ふさぎと言う行動に陥りやすい。なかなか泣き止まない乳児をあやそうとする行動がエスカレートして激しいゆさぶりになり、乳幼児揺さぶれ症候群の発症につながることがある。

泣きによるゆさぶりや乳幼児揺さぶれ症候群を減らすため、「何をやっても泣き止まないときはその場を離れて、まず自分がリラックスしましょう」というメッセージを伝えることが考えられている。このために開発されている教育媒体による介入の効果がいくつかのランダム化比較対象試験によって検証されている。ここからさらに発展して、地域の中でこういった介入により、実際にゆさぶり割合が減少するか、乳幼児揺さぶれ症候群の発生が減少するか、という事を検証してゆくことが関心となる。このような中で、藤原らの研究によりゆさぶり行動の存在割合とその関連要因が明らかになった意義は大きい。

ゆさぶり行動をするなど、泣きストレスをより強く感じる集団を捉えることは、公衆衛生上重要である。それは乳幼児揺さぶれ症候群の予防介入を行うターゲットポピュレーションであると同時に、不適切養育のリスクファクターを多く持つ集団を捉えることができるため、不適切養育に対する有効な介入の手だてともなりうる。このようにして、包括的な児童虐待予防が可能となる。こうした介入のベースラインとして、ゆさぶり行動の存在割合を明らかにすることはサービス提供の政策決定の上でも有益な情報となる。

Clarifying the prevalence of the behavior of baby shaking among the population is of great importance. Attention should be paid to the behavior of shaking because failure to sooth infants who continue crying could lead to the behavior of violently shaking the baby, which could lead to the severe consequence known as shaken baby syndrome (SBS).^1^

SBS is a certain kind of maltreatment that can result in severe brain damage and is defined as excessive shaking that comes from anger triggered by the crying of an infant. SBS has been suggested to be closely related to infant crying because the peak ages of both are quite similar (between 2 and 3 months of age).

The brains of small infants are vulnerable to stimulation. Their nervous systems are also unguarded because the myelin sheath is too immature to protect the nerve fibers. Further, the nuchal muscles of infants are weak; their strength is barely sufficient to hold up their heads. Therefore, an infant’s head whips when an adult violently shakes them back and forth. Centrifugal force is generated by the rotary motion, and inertial force is generated by the abrupt turn, which causes a shift between the dura mater covering the brain and the brain parenchyma, resulting in rupture of the veins followed by subdural or subarachnoid hemorrhage. The brain bouncing against the inner wall of the skull can also result in crushing of the brain. Such brain parenchymal injuries can also cause cerebral edema. Infants referred to the hospital with SBS actually show prominent neural symptoms, such as consciousness disorder, convulsions, and numbness, with a mortality rate of 25%, which is extremely high among illnesses that may cause death during infancy.

One way of preventing SBS is to decrease the prevalence of shaking of infants. In order to evaluate the effectiveness of intervention strategies to reduce shaking, the baseline prevalence of shaking behavior should be assessed in the community. Fujiwara et al^2^ have shown the prevalence for the first time in Japan. They have also shown the risk factors for shaking of infants, which are similar to the factors associated with a lowered threshold of tolerance of infant crying. Having correct knowledge about crying is one way to increase tolerance of crying and to reduce parenting stress accordingly.

Crying among infants is known to be prominent during the 2nd or 3rd months of age across all cultures, after which it begins to decline in frequency. Some infants will not stop crying, regardless of soothing efforts. Especially in these 2 to 3 months, infants may cry without specific reasons; they sometimes cry violently, and nothing can be done to stop it. However, this type of crying is a normal phenomenon, which will settle down gradually. This unexpected, continuous, and helpless crying, characterized by a painful facial expression without real pain and by selective occurrence in the late evening and at night, is known as “twilight crying” and “infantile colic.”

If parents are unaware of these characteristics of crying, they may put considerable effort into stopping infants crying and become exhausted. Some parents may have more difficulty tolerating this type of crying. Those parents may be prone to shake infants and smother them because they are irritated and do not want to hear them cry.

One strategy to reduce shaking and SBS is to deliver a message to parents: “When a baby won’t stop crying despite your efforts, leave and make yourself relaxed first.” Educational materials developed for this purpose first instruct parents to put every effort into stopping a baby’s crying and then explain that there is still the possibility that babies sometimes will not stop crying. In this case, parents should calm themselves down by leaving where the infants are crying whenever they become irritated and never to shake the baby. Additionally, such educational materials recommend sharing this information with others who might care for small infants. The effect of this intervention has been evaluated in a randomized controlled trial,^3^^–^^5^ and results have shown that the intervention has significant positive effects on outcomes, such as increased knowledge about infant’s crying, longer duration of holding a baby when it is crying, harm of shaking a baby, sharing information, and not blaming themselves for the infant’s crying.

Although these effects have been demonstrated in a randomized controlled trial, further investigation is required to confirm whether this intervention in a general population actually reduces the prevalence of infant shaking and SBS. In this situation, it is of great significance that Fujiwara et al have shown the prevalence of shaking and its related factors.

In order to design effective evidence-based interventions, a theoretical process is needed to clarify how, what, where, and to whom this kind of intervention should be done. It is already clear that appropriate understanding of crying decreases the stress caused by crying, and the attitude derived from such knowledge might contribute to decrease not only the shaking behavior but also other various inappropriate parenting behaviors.

In addition to the fact that population data for shaking are important for political decision-making to provide comprehensive parenting interventions, shaking an infant in itself is an inappropriate parenting practice, so designing an intervention to decrease this behavior alone could have a substantial impact on population health. Promoting knowledge about infants crying could play a role in a comprehensive intervention not only for a target population at increased risk of shaking but also for the larger general population.

Understanding the characteristics of parents who have high stress due to infants crying is important from the viewpoint of public health. Identifying parents with low tolerance for crying could be an effective strategy to identify those prone to infant shaking. Identifying parents with a low tolerance for infant crying is not only useful for targeting a population for preventive intervention for SBS but may also reveal a group in the population with multiple risk factors for poor relationships with their infants. Therefore, targeting parents at increased risk of shaking behavior may also have a role in effective interventions against inappropriate parenting, thereby contributing to the comprehensive prevention of child maltreatment. Clarifying the baseline prevalence of shaking for these interventions may help further improve the effectiveness of such comprehensive interventions to prevent child maltreatment.

## ONLINE ONLY MATERIAL

Abstract in Japanese.
