# Supplementary material for: Prevalence of Infant Shaking Among the Population as a Baseline for Preventive Interventions
Source: J Epidemiol. 2016 Jan 5;26(1):2–3. doi: 10.2188/jea.JE20150321 (PMC4690734; doi:10.2188/jea.JE20150321)
Supplement: Abstract in Japanese. [file je-26-002-s001.pdf]

地域における乳児ゆさぶり行動の存在割合---予防介入のベースラインとして

加藤則子
十文字学園女子大学人間生活学部

乳児は生後 2～3 か月までは、特に理由もなく泣き、なにをやっても泣き止まないほど激しく泣くが、正常な経過であり、次第に収まっていく。こういった泣きに対して、より耐性を持ちがたい親は、イライラしたり、無理に泣き止まそうとしたりして、ゆさぶりや口ふさぎと言う行動に陥りやすい。なかなか泣き止まない乳児をあやそうとする行動がエスカレートして激しいゆさぶりになり、乳幼児揺さぶれ症候群の発症につながることがある。

泣きによるゆさぶりや乳幼児揺さぶれ症候群を減らすため、「何をやっても泣き止まないときはその場を離れて、まず自分がリラックスしましょう」というメッセージを伝えることが考えられている。このために開発されている教育媒体による介入の効果がいくつかのランダム化比較対象試験によって検証されている。ここからさらに発展して、地域の中でこういった介入により、実際にゆさぶり割合が減少するか、乳幼児揺さぶれ症候群の発生が減少するか、という事を検証してゆくことが関心となる。このような中で、藤原らの研究によりゆさぶり行動の存在割合とその関連要因が明らかになった意義は大きい。

ゆさぶり行動をするなど、泣きストレスをより強く感じる集団を捉えることは、公衆衛生上重要である。それは乳幼児揺さぶれ症候群の予防介入を行うターゲットポピュレーションであると同時に、不適切養育のリスクファクターを多く持つ集団を捉えることができるため、不適切養育に対する有効な介入の手だてともなりうる。このようにして、包括的な児童虐待予防が可能となる。こうした介入のベースラインとして、ゆさぶり行動の存在割合を明らかにすることはサービス提供の政策決定の上でも有益な情報となる。
